# Severe Hereditary Pyropoikilocytosis/Hereditary Elliptocytosis–Like Phenotype Associated With a Heterozygous *SPTA1* Variant in a Cambodian Girl: A Case Report

**DOI:** 10.1155/crh/9545062

**Published:** 2026-09-26

**Authors:** Kim Leanghay, Chin Soey, Meang Sovandos, Srey Viso, Chean Sophâl

**Affiliations:** ^1^ Department of Pediatric Hematology and Immunology, National Pediatric Hospital, Phnom Penh, Cambodia; ^2^ Department of Medical Laboratory, National Pediatric Hospital, Phnom Penh, Cambodia

**Keywords:** anemia, child, hereditary pyropoikilocytosis, red blood cell membrane disorders, *SPTA1* variant

## Abstract

**Background:**

Hereditary elliptocytosis (HE) is typically a mild or asymptomatic red blood cell membrane disorder characterized predominantly by elliptocytes, whereas hereditary pyropoikilocytosis (HPP) represents the severe end of the HE spectrum, with early‐onset hemolysis, marked poikilocytosis, red cell fragmentation, and pronounced membrane instability. We report a Cambodian girl with a severe HPP/HE‐like phenotype associated with a heterozygous *SPTA1* variant of uncertain significance, emphasizing the importance of integrating clinical, hematologic, morphologic, and molecular findings to support accurate diagnostic classification.

**Case Presentation:**

A 7‐year‐old Cambodian girl born to nonconsanguineous parents was referred for evaluation of chronic transfusion‐dependent hemolytic anemia that began in the neonatal period. She developed severe jaundice at 1 week of age, requiring intensive phototherapy, and required regular packed red blood cell transfusions from 2 weeks of age. Corticosteroid therapy was initiated when immune‐mediated hemolysis was suspected, modestly prolonging the transfusion interval before being discontinued because of Cushingoid features. Physical examination revealed pallor, scleral icterus, and mild splenomegaly. Laboratory evaluation showed severe anemia, markedly elevated red cell distribution width, an inappropriately low reticulocyte count for the degree of anemia, a negative direct antiglobulin test, normal G6PD activity, and red cell morphological abnormalities, including poikilocytes, elliptocytes, and red cell fragments. Phenotype‐guided clinical whole‐exome sequencing identified a heterozygous *SPTA1* c.179G > C (p.Arg60Pro) variant of uncertain significance. The clinical presentation and red cell morphology supported classification as a severe HPP/HE‐like red cell membrane phenotype, with the heterozygous *SPTA1* variant considered an associated molecular finding rather than evidence of causality.

**Conclusion:**

This case illustrates the diagnostic complexity of severe HPP/HE‐spectrum disease associated with a heterozygous *SPTA1* variant. In children with early‐onset transfusion‐dependent hemolytic anemia and compatible red cell morphology, integrated clinical, hematologic, and molecular assessment, supported by family studies, membrane testing, and broader genomic analysis, may improve diagnostic classification when available.

## 1. Introduction

Hereditary pyropoikilocytosis (HPP) is a rare red blood cell membrane disorder and is considered a severe phenotype within the hereditary elliptocytosis (HE) spectrum [1, 2]. It typically presents in infancy or early childhood with chronic hemolytic anemia, marked poikilocytosis, red cell fragmentation, and, in severe cases, transfusion dependence [3]. The underlying mechanism involves instability of the red blood cell membrane cytoskeleton, particularly disruption of spectrin‐based horizontal interactions. Defective *α*‐ and *β*‐spectrin self‐association reduces membrane elasticity and increases red cell fragmentation, leading to chronic hemolysis [4–6].

HPP is usually associated with defects affecting both spectrin alleles, including homozygous or compound heterozygous variants in spectrin alpha, erythrocytic 1 *(SPTA1)*, or a heterozygous *SPTA1* variant combined with a low‐expression allele. Low‐expression *SPTA1* alleles, including *αLELY* and *αLEPRA*, may reduce *α*‐spectrin production and exacerbate disease severity when coinherited with a pathogenic variant. Therefore, the presence of a severe HPP/HE‐like phenotype in a patient with only a heterozygous *SPTA1* variant presents a diagnostic challenge and raises the possibility of undetected modifying alleles or additional genetic factors contributing to disease severity [1, 4, 5, 7, 8].

We report a Cambodian girl with lifelong transfusion‐dependent hemolytic anemia, HPP/HE‐spectrum morphology, and a heterozygous *SPTA1* c.179G > C (p.Arg60Pro) variant classified as a variant of uncertain significance. This case highlights the need to integrate clinical, morphologic, and molecular findings when confirmatory membrane studies and comprehensive genetic testing are unavailable.

## 2. Case Presentation

### 2.1. Clinical History

A 7‐year‐old Cambodian girl born to nonconsanguineous parents was referred for evaluation of chronic transfusion‐dependent hemolytic anemia. She was delivered by cesarean section at 39 weeks’ gestation to a healthy 24‐year‐old mother after a pregnancy complicated by oligohydramnios. Her birth weight was 2300 g. She was the second child in the family, and the mother’s first pregnancy had ended in miscarriage. Both parents were reportedly healthy, with no known family history of hemolytic anemia or a similar transfusion‐dependent disorder. Parental hematologic evaluation and segregation testing were not performed.

The patient developed severe neonatal jaundice at 1 week of age, requiring intensive phototherapy. From 2 weeks of age, she required packed red blood cell transfusions approximately every 2–4 weeks because of recurrent pallor and severe anemia. Corticosteroid therapy was initiated at 12 months of age, before the definitive diagnosis, when immune‐mediated hemolysis was considered, and the transfusion interval reportedly increased temporarily to approximately 6–12 weeks. However, treatment was discontinued because of Cushingoid features. Physical examination revealed pallor and scleral icterus without dysmorphic features; the spleen was palpable 3 cm below the left costal margin, and developmental milestones were normal.

### 2.2. Diagnostic Assessment

Initial laboratory evaluation showed severe anemia, with a hemoglobin level of 5.0 g/dL and hematocrit of 18%. Red cell distribution width was markedly elevated. Although the reticulocyte count was within the laboratory reference range at 0.5%, it was inappropriately low for the degree of anemia. Mean corpuscular volume was 85 fL, mean corpuscular hemoglobin concentration (MCHC) was 32 g/dL, and platelet count was 217 × 10^9^/L. Total and indirect bilirubin levels were mildly elevated at 1.71 mg/dL and 1.28 mg/dL, respectively. Urinary urobilinogen and the direct antiglobulin test were negative, and G6PD activity was normal. Serum ferritin was 279 ng/mL at the current evaluation, warranting continued surveillance because of cumulative transfusion exposure (Table 1).

**TABLE 1:** Serial laboratory results during follow‐up.

Blood work	Date of blood work				Reference range
	16‐06‐2025	11‐08‐2025	13‐10‐2025	12‐11‐2025	
*Complete blood count*		Results			
White blood cells	15.6	8.5	8.4	3.4	5–15 × 10^9^/L
Neutrophils	4.06	4.84	4.54	1.53	1.5–8 × 10^9^/L
Lymphocytes	9.98	3.06	3.28	1.77	6–9 × 10^9^/L
Red blood cells	2.10	2.89	2.58	3.11	4–5.2 × 10^12^/L
Hemoglobin	5.0 (↓)	7.2 (↓)	6.2 (↓)	7.9 (↓)	11–14 g/dL
Hematocrit	18 (↓)	22 (↓)	20 (↓)	24 (↓)	34%–40%
Reticulocytes	0.5	—	—	—	0.2%–2%
MCV	85	77	76	81	75–87 fL
MCHC	32	32	32	32	31–37 g/dL
Platelet count	217	258	314	193	200–490 × 10^9^/L
RDW‐CV	36 (↑)	39 (↑)	40 (↑)	30 (↑)	11.5%–14%

***Peripheral smear***	**Anisopoikilocytosis, poikilocytes, elliptocytes, red cell fragments**				

*Liver enzymes*					
AST	25	—	—	—	< 31 U/L
ALT	18	—	—	—	< 32 U/L
*Renal functions*					
Urea	22	—	—	—	10–50 mg/dL
Creatinine	0.6	—	—	—	0.5–0.9 mg/dL
*Biochemistry*					
Total bilirubin	1.71	—	—	—	0.3–1 mg/dL
Indirect bilirubin	1.28	—	—	—	0.2–0.8 mg/dL
Serum ferritin	279	—	—	—	27–375 ng/mL
G6PD	Normal	—	—	—	
*Immuno-hematology*					
Red blood type	A, Rh (+)	—	—	—	
Direct antiglobulin test	Negative	—	—	—	
*Special investigations*					
Osmotic fragility test	Not available				
PRC transfusion	Yes^∗^	Yes^∗^	Yes^∗^	Yes^∗^	

*Note:* ALT, alanine aminotransferase; AST, aspartate aminotransferase; RDW‐CV: red cell distribution width–coefficient of variation.

Abbreviations: G6PD, glucose‐6‐phosphate dehydrogenase; MCHC, mean corpuscular hemoglobin concentration; MCV, mean corpuscular volume; PRC, packed red cell.

^∗^Laboratory investigations were performed before packed red blood cell transfusion.

**FIGURE 1:**
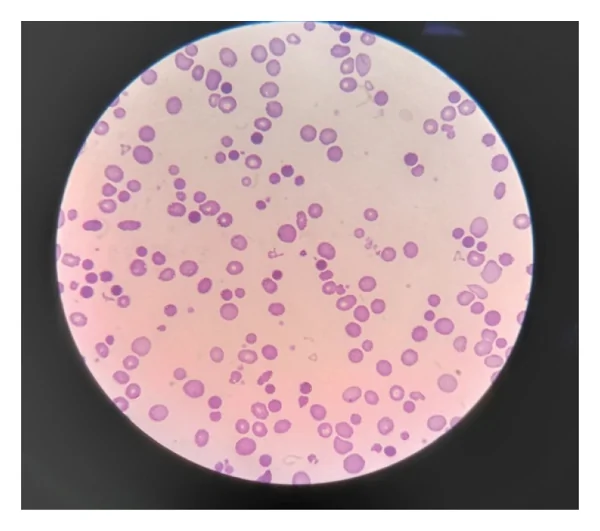
Peripheral blood smear obtained approximately 10 weeks after the preceding transfusion, showing marked anisopoikilocytosis (3+), elliptocytes/ovalocytes (2+), occasional red cell fragments or schistocyte‐like forms (1+), and absent‐to‐rare microspherocytes.

The blood sample and peripheral smear were obtained immediately before a scheduled packed red blood cell transfusion, approximately 10 weeks after the preceding transfusion (Figure 1).

After written parental consent, phenotype‐guided clinical whole‐exome sequencing identified a heterozygous *SPTA1* c.179G > C (p.Arg60Pro; rs750863150) variant. The reporting laboratory classified it as a variant of uncertain significance and noted the same classification in ClinVar. According to the report, the global allele frequency was not reported in gnomAD, and the variant was absent from the laboratory’s internal database of 420 Thai individuals. The report provided no functional or segregation evidence and cited no published case‐level evidence establishing causality; therefore, the variant’s contribution to the phenotype remains uncertain.

Further analysis of key anemia‐related genes identified no additional reportable pathogenic or likely pathogenic variants. However, exome sequencing may miss large deletions, copy‐number or structural variants, mosaicism, deep intronic or regulatory variants, and changes in difficult genomic regions; assessment of *αLELY* and *αLEPRA* was not documented.

Taken together, the early‐onset severe hemolytic anemia, lifelong transfusion dependence, and peripheral blood smear abnormalities supported classification as a severe HPP/HE‐like red cell membrane phenotype. The heterozygous *SPTA1* p.Arg60Pro variant was considered an associated molecular finding rather than diagnostic evidence, and an undetected modifying allele or other genomic contributor could not be excluded.

### 2.3. Treatment and Follow‐Up

The patient is currently managed with folic acid and multivitamin supplementation, together with symptom‐ and hemoglobin‐guided packed red blood cell transfusions. During follow‐up, she remained clinically stable with supportive management and had not undergone splenectomy. Ongoing surveillance for transfusional iron overload was planned. The clinical course, diagnostic evaluation, and management are summarized in Table 2.

**TABLE 2:** Timeline of clinical course, diagnostic evaluation, and management.

Age	Clinical event
Birth	Born at 39 weeks’ gestational age by cesarean section, birth weight 2300 g.

1 week	Developed severe neonatal jaundice requiring intensive phototherapy.

2 weeks	Initiated packed red blood cell (PRC) transfusion.

12 months	Corticosteroid therapy initiated when immune‐mediated hemolysis was suspected; subsequently discontinued because of Cushingoid features.

1–7 years	Regular PRC transfusions every 2–4 weeks because of severe anemia.

7 years	•Clinical whole‐exome sequencing with phenotype‐guided analysis identified a heterozygous SPTA1 c.179G > C (p.Arg60Pro) variant of uncertain significance. •Referred to National Pediatric Hospital for evaluation of chronic transfusion‐dependent hemolytic anemia.

Current	Folic acid and multivitamin supplementation, symptom‐ and hemoglobin‐guided PRC transfusions, and iron‐overload surveillance.

## 3. Discussion

HPP represents the severe end of the HE spectrum and is commonly associated with biallelic spectrin defects, particularly involving *SPTA1,* or with a disease‐associated *SPTA1* variant coinherited with a low‐expression allele [1, 5, 7–9]. These abnormalities impair spectrin‐based cytoskeletal interactions, reduce red cell membrane stability, and promote fragmentation and chronic hemolysis [2, 4–6].

The genotype–phenotype correlation in this patient remains uncertain. The identified p.Arg60Pro variant was classified as a variant of uncertain significance and does not independently establish causality. Although no additional reportable pathogenic or likely pathogenic variant was detected in the phenotype‐guided exome analysis, an undetected low‐expression *SPTA1* allele, deep intronic or regulatory variant, copy‐number or structural variant, or an additional red cell disorder gene could theoretically modify disease severity. However, these possibilities remain hypothetical and were not demonstrated in this patient.

An important consideration in this case is the differential diagnosis among red cell membrane disorders, particularly hereditary spherocytosis (HS) and Southeast Asian ovalocytosis (SAO). HS is usually associated with spherocytes and increased MCHC, reflecting membrane loss and cellular dehydration [2, 7, 10]. In contrast, MCHC in HPP is typically normal, as observed in our patient, or only mildly elevated, reflecting cytoskeletal instability. SAO, caused by mutations in *SLC4A1*, is characterized by rigid ovalocytes on peripheral blood smear, with MCHC values that are usually normal or only slightly increased [2]. Therefore, differentiation requires integration of red cell indices, peripheral smear morphology, clinical severity, and genetic findings. In this patient, the early‐onset severe anemia, transfusion dependence, marked anisopoikilocytosis, elliptocytes, and red cell fragments favored an HPP/HE‐like phenotype over HS or SAO. The heterozygous SPTA1 variant was considered an associated molecular finding rather than diagnostic evidence.

The reticulocyte count was inappropriately low for the degree of anemia. The sample was obtained immediately before a scheduled transfusion, approximately 10 weeks after the preceding transfusion. Residual donor erythrocytes and transfusion‐related suppression of endogenous erythropoiesis may have influenced the result, while an impaired or ineffective erythropoietic response cannot be excluded [5, 7]. Given her cumulative transfusion exposure, serial monitoring for iron overload and related long‐term complications is essential. Although endocrine complications are better characterized in chronically transfused hemoglobinopathies, iron accumulation may also contribute to endocrine morbidity in other transfusion‐dependent disorders [11]. Prospective multicenter studies are needed to clarify these long‐term outcomes in rare hereditary red cell membrane disorders.

The temporary reduction in transfusion requirement during corticosteroid therapy is unusual, as steroids are not standard treatment for HPP or HE. The mechanism is uncertain, and the development of Cushingoid features supports avoiding long‐term corticosteroid therapy unless another steroid‐responsive process is identified.

Management of severe HPP/HE‐like phenotypes is generally supportive and includes transfusion support, folic acid supplementation, and monitoring for iron overload. Splenectomy has been documented to reduce transfusion dependency and improve anemia in selected patients, although outcomes are variable. Both subtotal and total splenectomy have been described, with differing degrees of clinical benefit [3, 7]. If splenectomy is considered in the future, complete immunization against encapsulated organisms, including *Streptococcus pneumoniae*, *Haemophilus influenzae* Type B, and *Neisseria meningitidis*, should be ensured to reduce the risk of postsplenectomy infection [12].

Genetic counseling should address the uncertain molecular finding, possible inheritance patterns, testing limitations, and the potential contribution of additional genetic factors to future pregnancies and family planning.

## 4. Limitations

This report has several limitations, including the unavailability of specialized red cell membrane studies such as osmotic fragility testing, eosin‐5‐maleimide binding, ektacytometry, membrane‐protein analysis, and thermal sensitivity testing. Parental segregation testing and formal parental hematologic evaluation were not performed. Although clinical whole‐exome sequencing was conducted, the report lacked target‐level coverage metrics and individual ACMG/AMP criteria. Exome sequencing may also miss copy‐number changes, structural variants, deep intronic or regulatory variants, mosaic variants, and alterations in repetitive, high‐GC, or homologous regions. Specific assessment of the modifying SPTA1 alleles αLELY and αLEPRA was not documented, and whole‐genome sequencing, RNA analysis, and functional studies were not performed.

## 5. Conclusion

This case highlights the diagnostic complexity of a severe HPP/HE‐like phenotype in a Cambodian child with lifelong transfusion‐dependent hemolytic anemia and a heterozygous *SPTA1* c.179G > C (p.Arg60Pro) variant classified as a variant of uncertain significance. The variant does not establish causality in the absence of functional evidence, parental segregation studies, comprehensive detection of genomic or modifying variants, and confirmatory red cell membrane investigations. Clinical features, blood smear morphology, and molecular findings should therefore be interpreted together. Complementary testing, including family studies, specialized membrane assays, and broader genomic analysis, may improve diagnostic classification when available.

## Author Contributions

Kim Leanghay and Chean Sophâl conceptualized the case report and drafted the manuscript. Chin Soey and Meang Sovandos provided critical review and intellectual input. Srey Viso contributed to peripheral blood smear interpretation. Chean Sophâl edited and finalized the manuscript.

## Funding

This research received no specific grant from any funding agency in the public, commercial, or not‐for‐profit sectors.

## Disclosure

All authors have read and approved the final version of the manuscript. Kim Leanghay, the corresponding author and manuscript guarantor, had full access to all clinical data and takes complete responsibility for their integrity and accurate interpretation.

## Ethics Statement

According to institutional policy, formal ethical approval was not required for this retrospective descriptive case report.

## Consent

Written informed consent for publication of clinical details and accompanying images was obtained from the patient’s parents.

## Conflicts of Interest

The authors declare no conflicts of interest.

## Patient Perspective

The patient’s parents reported that the genetic evaluation helped them better understand the possible basis of their daughter’s condition and the importance of long‐term follow‐up.

## Supporting Information

Additional supporting information can be found online in the Supporting Information section.

## Supporting information


**Supporting Information 1** Clinical whole‐exome sequencing (cWES) report. It contains the complete molecular diagnostic analysis for a 7‐year‐old female patient evaluated for congenital hemolytic anemia. The document includes variant interpretation for the SPTA1 gene (c.179G > C, p.Arg60Pro) associated with hereditary pyropoikilocytosis.

**Supporting Information 2** CARE checklist—HPP HE case report.

## Data Availability

The data that support the findings of this study are available from the corresponding author upon reasonable request.
