# Supplementary material for: Severe Hereditary Pyropoikilocytosis/Hereditary Elliptocytosis–Like Phenotype Associated With a Heterozygous SPTA1 Variant in a Cambodian Girl: A Case Report
Source: Case Rep Hematol. 2026 Sep 26;2026:9545062. doi: 10.1155/crh/9545062 (PMC13615516; doi:10.1155/crh/9545062)
Supplement: Supplementary file 1 [file CRH-2026-9545062-s001.pdf]

Personal Report

This report is exclusively for

ATGenes

The World Class Biotechnology Company

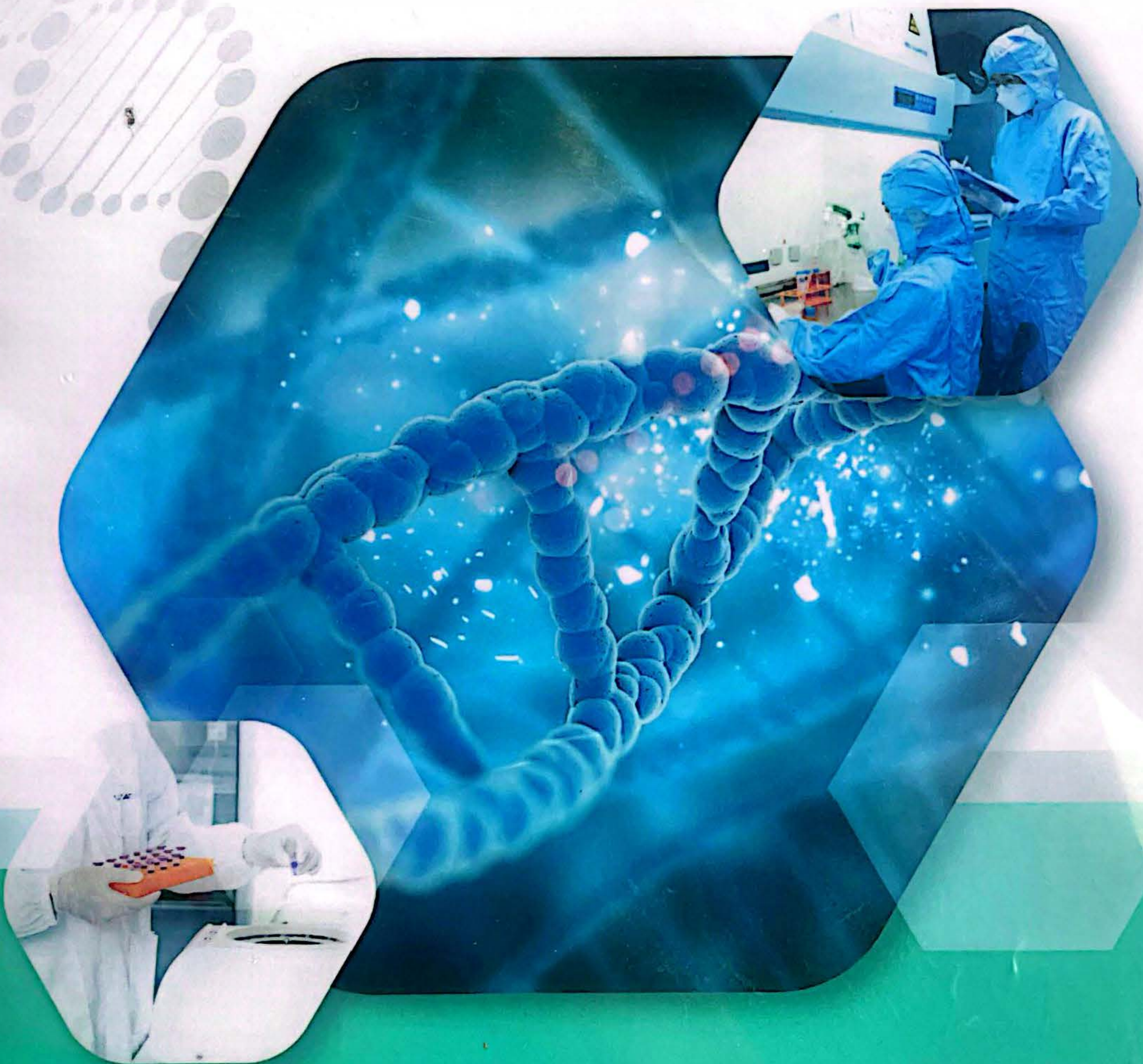

Clinical Whole Exome Sequencing (cWES)

This report is exclusively for :

DOB 12/06/2017 Age: 7 [illegible] Sex: Female

ATGenes

ATGenes, Co. Ltd.

8 Phutthamonthon Sai 1, Soi 37 Chimphli,
Khet Taling Chan, Bangkok 10170

Scanned with CamScanner

Date of birth: **12/06/2017** Ethnicity: - Sex: **Female**

Age: **7 years**

E-mail: **-**

Clinical diagnosis/Clinical surveillance for: **Congenital Hemolytic Anemia.**

HN: **1009406**

Date received: **05/07/2024**

Analyzing date: **12/09/2024**

Data Analysis: **Anemia HP:0001903**

Capture Kit: **SureSelect V6+UTR-post** Type of Sequencer: **Illumina platform**

Result Summary

Gene	Variant	Associated disorder	Zygosity	Classification
SPTA1	c.179G>C (p.Arg60Pro)	- Hereditary Pyropoikilocytosis (OMIM:266140)	Heterozygous	Uncertain significance

Result Interpretation

DNA sample from the patient underwent Whole Exome Sequencing and data analysis for a selected panel of genes related to clinical features. We found a heterozygous **c.179G>C** (rs750863150) missense variant in ***SPTA1*** gene which is related to Hereditary Pyropoikilocytosis (OMIM:266140). This mutation was reported as **Uncertain significance** in ClinVar. The global allele frequency is not reported in gnomAD. Hereditary Pyropoikilocytosis (OMIM:266140) is inherited in an autosomal recessive pattern of inheritance. Furthermore, by adjusting the analysis workflow, not to limit the allele frequency of any found variant, the result showed no additional pathogenic variants related to the clinical phenotype mentioned above.

SPTA1 gene (spectrin alpha chain, erythrocytic 1) encodes one of the two subunits of the spectrin protein, which is a crucial component of the cytoskeleton in red blood cells. Mutations in the ***SPTA1*** gene disrupt spectrin function, causing red blood cell abnormalities that result in severe anemia due to the production of abnormally shaped red blood cells. The abnormality in alpha-spectrin production could lead to Hereditary Pyropoikilocytosis. Additionally, the ***SPTA1*** **c.179G>C** variant is not found in our database (420 Thais).

SPTA1 mutations are significantly prevalent among Southeast Asians, including Thai population (Ittiwut et al., 2019 and Munkongdee et al.,2021). While specific data on the **c.179G>C** variant in Thai individuals is limited, the presence of other ***SPTA1*** mutations suggests a genetic predisposition to Hereditary Pyropoikilocytosis in this population. This information is valuable for genetic counselling, aiding in family planning decisions and potentially prompting further genetic testing for relatives.

Scanned with CamScanner

Further screening for possible deletions in ***SPTA1*** gene, and genes related to anemia, including ***SLC4A1, SPTB, EPB41, EPB42, HBA1, HBA2, HBB, HBD, HBG1, HBG2, HBZ, ALAS2*** and ***GATA1,*** additional significant deletions or mutation, including single nucleotide polymorphisms (SNPs) or indel variations were not identified. However, due to technical limitations in analyzing exome data, the detection of large deletions may be limited. Please correlate these results with other clinical information.

Further analysis based on this WES dataset is available free-of-charge depending on the clinician's discretion. Please contact your service provider; AThenaAI Co., Ltd. for further information.

Scanned with CamScanner

Appendix 2: Summary of all SNVs with HIGH and MODERATE Putative Impact on selected gene(s) panels

CHROM	POS	REF	ALT	QUAL	DP	AD	Zygosity	Effect	Impact	Gene	HGVS.c	HGVS.p	RS_NUMBER	1KG_AF	SIFT_pred	CLNSIG	OMIM
chr1	158685193	C	G	697.77	44	23	HET	missense variant	MODERATE	SPTA1	c.179G>C	p.Arg60Pro	rs750863150	.	D;.	Uncertain significance	Pyropoikilocytosis, hereditary (OMIM:266140)
chr14	94314431	G	A	1015.77	96	46	HET	missense variant	MODERATE	SERPINA6	c.218C>T	p.Ala73Val	rs146744332	0.003994	D	Not report in Clinvar	Corticosteroid-binding globulin deficiency (OMIM:611489)
chr8	143934039	A	T	560.77	45	27	HET	missense variant	MODERATE	PLEC	c.1633T>A	p.Cys545Ser	.	.	D	Not report in Clinvar	Epidermolysis bullosa simplex with muscular dystrophy (OMIM:226670)/ Epidermolysis bullosa simplex, Ogna type (OMIM:131950)/ Epidermolysis bullosa, junctional, with pyloric atresia (OMIM:226730)/ Muscular dystrophy, limb-girdle, type 2Q (OMIM:613723)/ Epidermolysis bullosa simplex with pyloric atresia (OMIM:612138)/ Epidermolysis bullosa simplex with nail dystrophy (OMIM:616487)/ Epidermolysis bullosa simplex with pyloric atresia (ORPHA:158684)/ Epidermolysis bullosa simplex with muscular dystrophy (ORPHA:257)/ Aplasia cutis congenita (ORPHA:1114)/ Plectin-related limb-girdle muscular dystrophy R17 (ORPHA:254361)/ PLEC-related intermediate epidermolysis bullosa simplex without extracutaneous involvement (ORPHA:79401)
chr9	69013924	G	C	1581.77	112	60	HET	missense variant	MODERATE	PRKACG	c.169C>G	p.Arg57Gly	.	.	D	Not report in Clinvar	Bleeding disorder, platelet-type, 19 (OMIM:616176)
chr6	32589669	G	C	1220.77	89	34	HET	missense variant	MODERATE	HLA-DRB1	c.74C>G	p.Pro25Arg	rs148093782	.	.;D	Not report in Clinvar	Sarcoidosis, susceptibility to, 1 (OMIM:181000)/ Multiple sclerosis, susceptibility to (OMIM:126200)/ Sarcoidosis (ORPHA:797)/ Diffuse cutaneous systemic sclerosis (ORPHA:220393)/ Giant cell arteritis (ORPHA:397)/ Autoimmune pulmonary alveolar proteinosis (ORPHA:747)/ Limited cutaneous systemic sclerosis (ORPHA:220402)/ Systemic-onset juvenile idiopathic arthritis (ORPHA:85414)/ Narcolepsy type 1 (ORPHA:2073)/ Narcolepsy type 2 (ORPHA:83465)/ Follicular lymphoma (ORPHA:545)/ Bullous pemphigoid (ORPHA:703)
chr6	32580779	C	T	100.77	32	5	HET	missense variant	MODERATE	HLA-DRB1	c.730G>A	p.Ala244Thr	rs3830125	.	.;D	Not report in Clinvar	Sarcoidosis, susceptibility to, 1 (OMIM:181000)/ Multiple sclerosis, susceptibility to (OMIM:126200)/ Sarcoidosis (ORPHA:797)/ Diffuse cutaneous systemic sclerosis (ORPHA:220393)/ Giant cell arteritis (ORPHA:397)/ Autoimmune pulmonary alveolar proteinosis (ORPHA:747)/ Limited cutaneous systemic sclerosis (ORPHA:220402)/ Systemic-onset juvenile idiopathic arthritis (ORPHA:85414)/ Narcolepsy type 1 (ORPHA:2073)/ Narcolepsy type 2 (ORPHA:83465)/ Follicular lymphoma (ORPHA:545)/ Bullous pemphigoid (ORPHA:703)

Scanned with CamScanner

Appendix 3: Schematic diagram showing Whole Exome Sequencing (WES) analytical process

Limitations of the exome sequencing

Exome sequencing does not target 100% of the genes in the human genome; approximately 97% of exons are targeted. However, ~10% of exons may not be covered at sufficient levels to reliably call heterozygous variants. Each individual may have slightly different coverage yield distributions across the exome. Clinical sensitivities and specificities of any individual exome are not calculated. Rare variants at the probe target site may affect analytical sensitivity.

Exome sequencing is limited in detecting the following types of mutations (this list might not be exhaustive):

- large rearrangements
- copy number variation mutations (large deletions/duplications)
- mitochondrial genome mutations
- epigenetic factors
- mosaic mutations
- uniparental disomy
- mutations in repetitive or high GC rich regions
- mutations in genes with corresponding pseudogenes or other highly homologous sequences

Ref: https://www.asperbio.com/ngs-service/limitations-of-the-exome-sequencing/

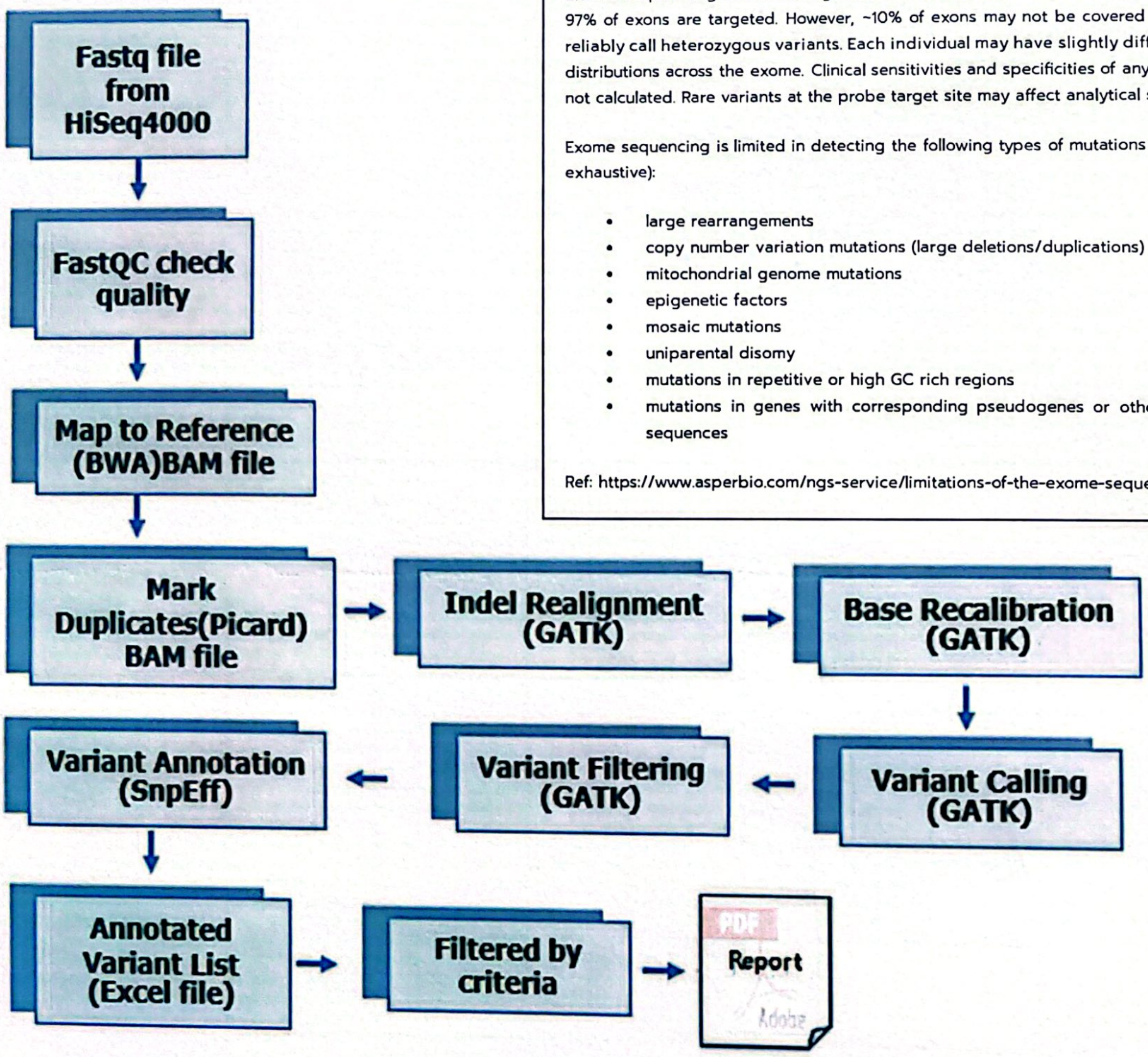

Summary

Total reads	68,042,170	(100%)
Mapped reads to human genome	67,965,445	(99.9%)
Unmapped reads to human genome	76,725	(00.1%)

Copyright©: reserved to ATGenes Co., Ltd. Advanced Molecular Genetic Laboratory ATGenes Company Ltd., Bangkok 10170, Thailand Tel: +66-2-073-1411-2

Scanned with CamScanner
