# Supplementary material for: Severe Hereditary Pyropoikilocytosis/Hereditary Elliptocytosis–Like Phenotype Associated With a Heterozygous SPTA1 Variant in a Cambodian Girl: A Case Report
Source: Case Rep Hematol. 2026 Sep 26;2026:9545062. doi: 10.1155/crh/9545062 (PMC13615516; doi:10.1155/crh/9545062)
Supplement: Supplementary file 2 [file CRH-2026-9545062-s002.pdf]

CARE
case report guidelines

CARE Checklist of information to include when writing a case report

(cc) BY-NC-ND

Topic	Item	Checklist item description	Reported on Line
Title	1	The diagnosis or intervention of primary focus followed by the words "case report"	p. 1
Key Words	2	2 to 5 key words that identify diagnoses or interventions in this case report, including "case report"	p. 1
Abstract (no references)	3a	Introduction: What is unique about this case and what does it add to the scientific literature?	p. 1
	3b	Main symptoms and/or important clinical findings	p. 1
	3c	The main diagnoses, therapeutic interventions, and outcomes	p. 1
	3d	Conclusion—What is the main "take-away" lesson(s) from this case?	p. 1
Introduction	4	One or two paragraphs summarizing why this case is unique (**may include references**)	p. 2
Patient Information	5a	De-identified patient specific information.	p. 2
	5b	Primary concerns and symptoms of the patient.	p. 2
	5c	Medical, family, and psycho-social history including relevant genetic information	pp. 2, 4
	5d	Relevant past interventions with outcomes	pp. 2, 4-5
Clinical Findings	6	Describe significant physical examination (PE) and important clinical findings.	p. 2
Timeline	7	Historical and current information from this episode of care organized as a timeline	p. 5
Diagnostic Assessment	8a	Diagnostic testing (such as PE, laboratory testing, imaging, surveys).	pp. 2-4
	8b	Diagnostic challenges (such as access to testing, financial, or cultural)	pp. 4, 6
	8c	Diagnosis (including other diagnoses considered)	pp. 4-5
	8d	Prognosis (such as staging in oncology) where applicable	N/A
Therapeutic Intervention	9a	Types of therapeutic intervention (such as pharmacologic, surgical, preventive, self-care)	pp. 2, 4-5
	9b	Administration of therapeutic intervention (such as dosage, strength, duration)	pp. 2, 4-5
	9c	Changes in therapeutic intervention (with rationale)	pp. 2, 5
Follow-up and Outcomes	10a	Clinician and patient-assessed outcomes (if available)	pp. 2, 4-5
	10b	Important follow-up diagnostic and other test results	pp. 3-4
	10c	Intervention adherence and tolerability (How was this assessed?)	pp. 2, 4-5
	10d	Adverse and unanticipated events	pp. 2, 4-5
Discussion	11a	A scientific discussion of the strengths AND limitations associated with this case report	p. 6
	11b	Discussion of the relevant medical literature **with references**.	pp. 2, 5-6
	11c	The scientific rationale for any conclusions (including assessment of possible causes)	pp. 4-6
	11d	The primary "take-away" lessons of this case report (without references) in a one paragraph conclusion	p. 6
Patient Perspective	12	The patient should share their perspective in one to two paragraphs on the treatment(s) they received	p. 7
Informed Consent	13	Did the patient give informed consent? Please provide if requested	Yes ☒ No ☐
